# Psychological Capital Mediates the Relationship Between Problematic Smartphone Use and Learning Burnout in Chinese Medical Undergraduates and Postgraduates: A Cross-Sectional Study

**DOI:** 10.3389/fpsyg.2021.600352

**Published:** 2021-05-13

**Authors:** Changhong Zhang, Ge Li, Zhaoya Fan, Xiaojun Tang, Fan Zhang

**Affiliations:** ^1^School of Public Health and Management, Research Center for Medicine and Social Development, Collaborative Innovation Center of Social Risks Governance in Health, Chongqing Medical University, Chongqing, China; ^2^The Center of Experimental Teaching Management, Chongqing Medical University, Chongqing, China

**Keywords:** learning burnout, mobile phone addiction, psychological capital, mediating effect, medical students

## Abstract

Learning burnout is a pressing issue among Chinese medical undergraduates and Postgraduates and has drawn continuous attention worldwide. Studies have found that problematic smartphone use could affect learning burnout, but more research is needed in this direction. Furthermore, few studies focused on the mediating effect of psychological capital on the relationship between problematic smartphone use and learning burnout. The present study was a cross-sectional survey that recruited 1,800 participants from a medical university in Chongqing, China. A questionnaire based on the Mobile Phone Addiction Tendency Scale, Psychological Capital Questionnaire, Learning Burnout Scale, and demographic variables were administered to these students, and 1,475 provided valid responses (81.94%). 771 were undergraduates (52.3%) and 704 were postgraduates (47.7%). Hierarchical regression and the bootstrap method were used to examine the mediating effect of psychological capital. After controlling for demographic variables, problematic smartphone use positively predicted learning burnout in undergraduates (β = 0.328, *p* < 0.01) and in postgraduates (β = 0.342, *p* < 0.01). The partial mediating effect of psychological capital was 0.068 in undergraduates and 0.074 in postgraduates, accounting for 20.67 and 21.64%, respectively, of the total effect of problematic smartphone use on learning burnout. All the 95% confidence intervals (CI) did not contain 0. Problematic smartphone use can directly predict learning burnout and their relationship was mediated by psychological capital in Chinese medical undergraduates and postgraduates. Strategies to alleviating problematic smartphone use and enhance psychological capital in medical undergraduates and postgraduates may provide useful suggestions for future interventions on dealing with learning burnout in Chinese medical undergraduates and postgraduates.

## Introduction

The outbreak of COVID-19 at the end of 2019 demonstrated that medical workers are indispensable. China, the most populous country in the world, experienced a lack of doctors leading to a gap between health service demand and healthcare supply (Zhang et al., 2020). The doctor-patient ratio was 2.77 doctors for 1,000 patients in 2020 in China (National Bureau of Statistics of China, 2020), immensely lower than that in some developed countries (Wan et al., 2018). Over the past 10 years, the attrition rate of medical graduates and physicians in China has been high (Lien et al., 2016). Therefore, Chinese medical students have drawn continuous attention worldwide. Learning burnout is an urgent problem among Chinese medical students, with an incidence of about 50% (Jinghua et al., 2014) and a high-risk rate of up to 10% (Liu et al., 2018).

Doctors are required to be highly specialized, therefore medical students need to spend more time and energy acquiring the necessary professional knowledge and skills (Margaret et al., 2010); they are under more academic pressure than students in other majors (Bond et al., 2013; Zeng et al., 2019). Learning burnout is a phenomenon that refers to energy depletion and negative attitudes toward learning (Zhang et al., 2007). The enthusiasm for learning gradually fades away, and attitudes toward classmates become increasingly cold and alienated due to prolonged academic pressure (Wang et al., 2020). Learning burnout, also called student burnout or academic burnout syndrome, involves emotional exhaustion, depersonalization, and a low sense of achievement (Lin and Huang, 2012). Studies have found that learning burnout strongly affects the academic performance (Fiorilli et al., 2017), concentration (Xie et al., 2019), and physical and mental health of medical students; for example; learning burnout is associated with sleep disorders, anxiety, depression (Njim et al., 2019), loneliness, and interpersonal problems; and can even lead to suicide and withdrawal from school (Stockman, 2010). Therefore, for future interventions, it is important to investigate the variables related to learning burnout.

Problematic smartphone use, with an incidence of more than 35%, is another pressing matter among Chinese medical students (Jing et al., 2016). It’s a phenomenon that physical and mental health, and social functions are remarkably impaired because of obsession with mobile phones (Yen et al., 2009). Problematic smartphone use, also have been called mobile phone addiction, is an addiction behavior (Zou et al., 2017), indicating poor self-control skills, studies found that these behaviors were associated with learning burnout (Love et al., 2020). Indeed, it confirmed that problematic smartphone use risk was positively related to perceived stress and negatively related to academic performance (Samaha and Hawi, 2016), which may lead learning burnout of students. Few studies have attempted to explore the relationship between problematic smartphone use and learning burnout among Chinese college students, and it found that problematic smartphone use can positively predict learning burnout of college students (Sijia and Cancan, 2018), but more research is needed to explore the mechanism between this relationship, especially among Chinese medical students.

Psychological capital represents the development of positive psychology of people, resulting positive organizational behaviors and demonstrates performance (Costa and Neves, 2017); and it includes four dimensions: self-efficacy, hope, optimism, and resilience (Luthans et al., 2007a). Psychological capital is related to the achievements and well-being of individuals (Margaça et al., 2020), and it can be exploited like social resources (Luthans et al., 2006). Several studies have indicated that internet addiction is negatively correlated with psychological capital (Simsek and Sali, 2014). Problematic smartphone use also belongs to addiction behaviors (Zou et al., 2017), which also go against positive organizational behaviors and demonstrates performance. Study confirmed problematic smartphone use was positively associated with psychological factors, such as depression, anxiety, and loneliness (Darcin et al., 2015), and also associated with poor mental health (Contractor et al., 2017); psychological capital has a theoretical intervention model (Luthans et al., 2005), but mental health does not have, and mental health involves many factors, which is not conducive to follow-up targeted intervention aimed at learning burnout, so we assumed psychological capital rather than mental health as a mediating variable based on the exploitation of psychological capital. Mental health is positively correlated withpsychological capital. Therefore, students with problematic smartphone use are more likely to develop poor psychological capital. However, according to existing literatures, psychological capital may have an effect on learning burnout. Previous studies confirmed that psychological capital could affect performance (Luthans et al., 2005) and well-beings (Avey et al., 2010) and reduces burnout (Ding et al., 2015) among employees, such as nurses and bank clerks (Li et al., 2015). As positive psychological resources, psychological capital is highly likely to reduce burnout among Chinese medical students; also, poor psychological capital is highly likely to lead to bad academic performance, resulting learning burnout (Fiorilli et al., 2017).

**FIGURE 1:**
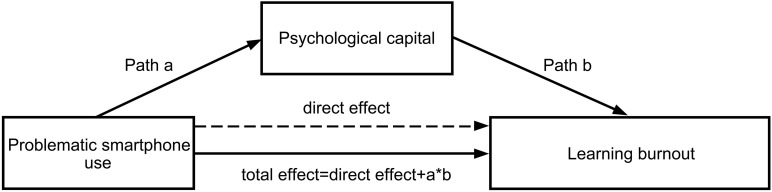
The hypothetical theoretical model; note: a^∗^b, indirect effect as well as the mediating effect of psychological capital in this relationship.

According to the above mentioned, and also according to the conditions for the establishment of the mediation model, namely, the independent variable must significantly affect both the dependent variable and mediating variables, and the mediating variable must have a significant effect on the dependent variable (Wen et al., 2004), we proposed the following hypotheses, namely, problematic smartphone use can directly predict learning burnout, and their relationship was mediated by psychological capital. The theoretical mediation model we hypothesized was shown in Figure 1.

This study aimed to analyze the relationship between problematic smartphone use and learning burnout, as well as the mediating effect of psychological capital in this relationship of Chinese medical students. Everyone has psychological resources at various levels, therefore, if the mediating effect of psychological capital once be confirmed, interventions might be conducted in the future to deal with learning burnout based on the exploitation of psychological capital. We hope that this study will contribute to providing clues for future interventions on dealing with learning burnout among Chinese medical student.

## Materials and Methods

### Participants

The study participants were undergraduate and postgraduate medical students, 771 were undergraduates (52.3%) and 704 were postgraduates (47.7%). The demographics of undergraduates are as follows: the average age was 19.87 ± 1.187; males (36.4%) and females (63.6%); students from countryside (35.5%) and 497 city (64.5%); only child (43.1%) and non-only child (56.9%). The demographics of postgraduates are as follows: the average age was 24.68 ± 2.45; males (27.7%) and females (72.3%); students from countryside (45.3%) and 497 city (54.7%); only child (47.9%) and non-only child (52.1%). The protocol and data collection procedure of the study were approved by the Chongqing Medical University (Reference number 2018015).

### Instruments

#### Mobile Phone Addiction Tendency Scale

The Mobile Phone Addiction Tendency Scale, developed by Jie (Jie et al., 2012) was used to measure mobile phone addiction or problematic smartphone use among Chinese college students. It has 16 items with four dimensions: withdrawal symptoms (six items; e.g., “Mobile phones are a part of my life. I feel like I’ve lost something once I will have to limit the time I spend on my mobile phone”), salience (four items; e.g., “I often have the illusion that ‘my cell phone rings or vibrates”), social comfort [three items; e.g., “I feel more confident to communicate with others using my mobile phone (than to talk face to face)”], and mood changes (three items; e.g., “When the phone is not connected to the line or receives no signals, I will become anxious and get angry”). All 16 items are rated on a five-point scale ranging from 1 (very inconsistent) to 5 (very consistent). Items were summed to create a composite score for each participant. A higher score indicates a greater tendency for mobile phone addiction. The total scores ranges from 16 to 80. In this study, Cronbach’s alpha was 0.900.

#### Psychological Capital Questionnaire

The Psychological Capital Questionnaire was created by Luthans et al. (2007b), translated and revised by Wen and Qi for Chinese individuals (Wen and Qi, 2009). The questionnaire consists of four dimensions, with six items each (24 items total): hope (e.g., “If I should find myself in a jam, I could think of many ways to get out of it”), resilience (e.g., “I usually manage difficulties one way or another during training”), optimism (e.g., “I always look on the bright side of things”), and self-efficacy (e.g., “I feel confident analyzing a long-term problem to find a solution”). Each item is rated on a six-point scale ranging from 1 (strongly disagree) to 6 (strongly agree). Items were summed to create a composite score for each participant, with higher scores indicating a higher level of psychological capital. The total score ranges from 24 to 144. A score of 100 or more indicates a good level of psychological capital. In this study, Cronbach’s alpha was 0.943.

#### Learning Burnout Questionnaire

The Learning Burnout Questionnaire was developed by Lian et al. (2005) and it was widely used to measure the learning burnout of Chinese college students. The questionnaire consists of 20 items including three dimensions: depression (eight items; e.g., “I feel exhausted after studying all day. I’m tired of studying”), improper behavior (six items; e.g., “I seldom study after class”), and low personal accomplishment (six items; e.g., “It is easy for me to master professional knowledge”). All 20 items are rated on a 5-point scale ranging from 1 (very inconsistent) to 5 (very consistent). The total score ranges from 20 to 100, and the mid-range is 60. Items were summed to create a composite score for each participant. A higher score indicates a greater perceived tendency to learning burnout. In this study, Cronbach’s alpha for the scale was 0.876.

### Procedure

A cross-sectional survey was conducted in the only one medical university in Chongqing, one of the biggest cities in China. Convenience sampling was used to generate the sample. After the researchers obtained informed consent from all participants, the latter completed a traditional paper-and-pencil questionnaire in a classroom with the guidance of well-trained researchers; averagely, it took about 30 min for participants to complete the anonymous questionnaire.

### Data Analyses

In this study, we analyzed undergraduate and postgraduate students separately because of their different psychosocial development stages. Descriptive analysis was used to describe the demographic characteristics of the participants. Enumeration data were described as percentages. Normally distributed continuous data were described as mean ± standard deviation. A *t*-test or one-way analysis of variance was used to analyze the differences in learning burnout (depression, improper behavior, and low personal accomplishment) among groups. The Pearson correlation coefficient was used to evaluate the corrections among the research variables. Hierarchical regression was used to explore the mediating effect of psychological capital on the relationship between problematic smartphone use and learning burnout (Zimet et al., 1990; Yang et al., 2020). The variance inflation factor (VIF) values were less than 10, and there was no multicollinearity in this study. In the hierarchical regression analysis, learning burnout and it’s three dimensions were took as dependent variables, respectively; in step 1, variables in univariate analysis (*p* < 0.05) were entered, in other words, these variables where learning burnout and it’s three dimensions were differences in *t*-test or one-way analysis were entered in step 1 to control their influences on the dependent variable, because our ultimate goal was to analyze the mediating effect of psychological capital; In step 2, the independent variable, problematic smartphone use, was entered, this step was to analyze it’s impact, and also to control its impact on the dependent variable for the next step. In step 3, psychological capital was entered as a mediating variable, this step was to analyze the effect of psychological capital on the dependent variable. We also set psychological capital as the dependent variable to analyze the effect of problematic smartphone use on it; then, we calculated the mediating effect value. Bootstrap method was used to examine the significance of the mediating effect of psychological capital. We bootstrapped 5,000 samples from the data, and calculated the 95% bootstrap confidence intervals (CI). If the CI did not contain 0, the mediating effect was considered significant (Wang et al., 2020). SPSS20.0 statistical software was used for statistical analysis, and the level of statistical significance was two-tailed *p* < 0.05.

## Results

### Demographic Data and the Analysis of Differences in Learning Burnout Among Variables

In undergraduates, the mean total score of the learning burnout was 56.42 ± 10.62, lower than the middle level; but 36.4% undergraduates had a score higher than the mid-value (60), indicating that they had a high level of learning burnout; there were significant differences in undergraduates’ total scores for learning burnout by gender (*t* = 2.446; *p* < 0.05), exercise per week (*F* = 11.466; *p* < 0.01) and attitude to majors (*F* = 68.111, *p* < 0.01). In postgraduates, the mean total score of the learning burnout was 53.92 ± 10.25; 27.6% undergraduates had a score higher than the mid-value (60), indicating that they had a high level of learning burnout; there were significant differences in undergraduates’ total scores for learning burnout by gender (*t* = −1.980; *p* < 0.05), exercise per week (*F* = 19.240; *p* < 0.01) and attitude to majors (*F* = 78.914; *p* < 0.01). There also had a significant difference in the mean total score of learning burnout between undergraduates and postgraduates (*t* = −4.591; *p* < 0.01). Detailed results are shown in Table 1.

**TABLE 1:** Demographic characteristics and comparisons on learning burnout and its three dimensions among undergraduates and postgraduates.

**Education background**	**Variables**	***n* (%)**	**Learning burnout (Mean ± SD)**	**Depression (Mean ± SD)**	**Improper behavior (Mean ± SD)**	**Low personal accomplishment (Mean ± SD)**
Undergraduates		771 (100)	56.42 ± 10.62^*a*^	22.19 ± 5.18	17.61 ± 4.03	16.61 ± 3.63
	Gender					
	Male	281 (36.4)	57.65 ± 10.96	22.83 ± 5.34	18.35 ± 4.31	16.47 ± 3.88
	Female	490 (63.6)	55.71 ± 10.36	21.83 ± 5.06	17.19 ± 3.80	16.69 ± 3.49
	*t*		2.446	2.598	3.754	−0.837
	*P*		0.015*	0.010*	0.000**	0.403
	Residence					
	Countryside	274 (35.5)	57.08 ± 10.80	22.28 ± 5.14	17.78 ± 4.27	17.03 ± 3.69
	City	497 (64.5)	56.05 ± 10.51	22.15 ± 5.22	17.52 ± 3.89	16.38 ± 3.59
	*t*		1.289	0.329	0.851	2.359
	*P*		0.198	0.741	0.395	0.019*
	Only child					
	Yes	332 (43.1)	55.94 ± 10.53	22.20 ± 5.29	17.37 ± 4.03	16.37 ± 3.69
	No	439 (56.9)	56.79 ± 10.68	22.19 ± 5.11	17.80 ± 4.02	16.79 ± 3.59
	*t*		−1.100	0.006	−1.465	−1.599
	*P*		0.272	0.995	0.143	0.110
	Exercise per week					
	0	227 (29.4)	59.13 ± 10.63	23.24 ± 5.19	18.72 ± 3.97	17.17 ± 3.84
	1∼3	461 (59.8)	55.51 ± 10.33	21.85 ± 5.02	17.21 ± 3.95	16.44 ± 3.46
	≥3	83 (10.8)	54.05 ± 10.88	21.23 ± 5.69	16.81 ± 4.08	16.01 ± 3.84
	*F*		11.466	7.194	12.908	4.306
	*P*		0.000**	0.001**	0.000**	0.014*
	Attitude to majors					
	Dislike	51 (6.6)	64.43 ± 10.51	25.73 ± 5.37	19.57 ± 4.19	19.14 ± 4.05
	Medium	438 (56.8)	58.78 ± 9.54	23.19 ± 4.64	18.21 ± 3.75	17.37 ± 3.27
	Like	282 (36.6)	51.31 ± 10.06	20.00 ± 5.13	16.34 ± 4.07	14.97 ± 3.44
	*F*		68.111	51.026	26.630	58.362
	*P*		0.000**	0.000**	0.000**	0.000**
Postgraduates		704 (100)	53.92 ± 10.25^*a*^	21.74 ± 5.16	16.64 ± 3.69	15.54 ± 3.15
	Gender					
	Male	195 (27.7)	52.69 ± 10.92	21.22 ± 5.32	16.56 ± 4.01	14.90 ± 3.23
	Female	509 (72.3)	54.39 ± 9.95	21.94 ± 5.09	16.67 ± 3.56	15.78 ± 3.09
	*t*		−1.980	−1.664	−0.347	−3.319
	*P*		0.048*	0.097	0.729	0.001**
	Residence					
	Countryside	319 (45.3)	53.71 ± 9.91	21.47 ± 5.04	16.71 ± 3.45	15.53 ± 3.08
	City	385 (54.7)	54.10 ± 10.53	21.97 ± 5.26	16.59 ± 3.87	15.54 ± 3.22
	*t*		−0.499	−1.261	0.414	−0.044
	*P*		0.618	0.208	0.679	0.965
	Only child					
	Yes	337 (47.9)	54.54 ± 10.99	22.05 ± 5.38	16.84 ± 3.90	15.65 ± 3.45
	No	367 (52.1)	53.35 ± 9.50	21.46 ± 4.95	16.46 ± 3.48	15.43 ± 2.97
	*t*		1.529	1.501	1.385	0.992
	*P*		0.127	0.134	0.167	0.357
	Exercise per week					
	0	299 (42.5)	55.78 ± 9.94	22.51 ± 4.99	17.25 ± 3.64	16.02 ± 3.06
	1∼3	306 (43.5)	52.68 ± 9.67	21.29 ± 5.04	16.30 ± 3.50	15.09 ± 3.04
	≥3	99 (14.1)	52.14 ± 11.99	20.85 ± 5.76	15.86 ± 4.13	15.43 ± 3.15
	*F*		19.240	6.018	7.696	6.779
	*P*		0.000**	0.003**	0.000**	0.001**
	Attitude to majors					
	Dislike	26 (3.7)	64.46 ± 9.53	26.50 ± 5.65	19.04 ± 3.30	18.92 ± 2.92
	Medium	363 (51.6)	57.20 ± 8.90	23.24 ± 4.64	17.44 ± 3.42	16.51 ± 2.86
	Like	315 (44.7)	49.28 ± 9.67	19.62 ± 4.83	15.52 ± 3.69	14.13 ± 2.85
	*F*		78.914	62.125	30.919	77.547
	*P*		0.000**	0.000**	0.000**	0.000**

^*a*^With the *t*-test, and there are significant differences between them (*t* = −4.591, *p* < 0.001); *p < 0.05, **p < 0.01.

### Correlation Among Psychological Capital, Problematic Smartphone Use, and Learning Burnout

The results showed that there were a moderate positive correlation between problematic smartphone use and learning burnout (*r* = 0.356; *p* < 0.01), a moderate negative correlation between problematic smartphone use and psychological capital (*r* = −0.205; *p* < 0.01), a moderate negative correlation between psychological capital and learning burnout (*r* = −0.532; *p* < 0.01) in undergraduates. In postgraduates, a moderate positive correlation between problematic smartphone use and learning burnout (*r* = 0.405; *p* < 0.01), a moderate negative correlation between problematic smartphone use and psychological capital (*r* = −0.219; *p* < 0.01), a moderate negative correlation between psychological capital and learning burnout (*r* = −0.594; *p* < 0.01) also existed. Detailed results are shown in Table 2.

**TABLE 2:** Correlation among psychological capital, problematic smartphone use, and learning burnout.

**Education background**	**Variables**	**Mean ± SD**	**1**	**2**	**3**	**4**	**5**	**6**
Undergraduates	1. PSU	41.39 ± 10.15	1					
	2. PC	98.79 ± 14.58	−0.205**	1				
	3. Depression	22.19 ± 5.18	0.382**	−0.405**	1			
	4. IB	17.61 ± 4.03	0.280**	−0.414**	0.635**	1		
	5. LPA	16.61 ± 3.63	0.210**	−0.516**	0.455**	0.436**	1	
	6. LB	56.42 ± 10.62	0.356**	−0.532**	0.885**	0.939**	0.730**	1
Postgraduates	1. PSU	42.63 ± 10.29	1					
	2. PC	101.24 ± 16.50	−0.219**	1				
	3. Depression	21.74 ± 5.16	0.417**	−0.474**	1			
	4. IB	16.64 ± 3.69	0.346**	−0.474**	0.690**	1		
	5. LPA	15.54 ± 3.15	0.230**	−0.598**	0.531**	0.486**	1	
	6. LB	53.92 ± 10.25	0.405**	−0.594**	0.915**	0.857**	0.750**	1

PSU, problematic smartphone use; PC, psychological capital; IB, improper behavior; LPA, low personal accomplishment; LB, learning burnout. *p < 0.05, **p < 0.01.

### Problematic Smartphone Use and Psychological Capital Are Independent Factors of Learning Burnout

The results of hierarchical regression analysis showed that after controlling for significant variables in the univariate analysis in step 1, for undergraduates, problematic smartphone use positively explained 9.3% of the variance of in learning burnout, for postgraduates, it was 11.5%; then, problematic smartphone use was controlled in step 2, for undergraduates, psychological capital negatively explained 13.7 of the variance in learning burnout, for postgraduates, it was 17.1. Besides, Problematic smartphone use was negatively predicted psychological capital by explaining 2.6% of its variance in undergraduates, and 2.0% in postgraduates. Detailed results are shown in Table 3. The analysis results of the three dimensions of learning burnout are consistent with that of learning burnout, detailed results are shown in Table 4.

**TABLE 3:** The effect of problematic smartphone use and psychological capital on learning burnout.

**Education background**	**Variables**	**Psychological capital**		**Learning burnout**		
		**Block1 (β)**	**Block2 (β)**	**Block1 (β)**	**Block2 (β)**	**Block3 (β)**
Undergraduates	Gender	0.287	0.204	−2.090**	−1.974**	−1.915**
	Exercise per week	2.504**	2.367**	−2.430**	−2.236**	−1.547**
	Attitude to majors	7.779**	7.264**	−6.663**	−5.934**	−3.820**
	Problematic smartphone use		−0.232**		0.328**	0.261**
	Psychological capital					−0.291**
	*R*^2^	0.116	0.141	0.173	0.270	0.407
	*F*	33.386	31.464	53.511**	70.710**	104.979**
	Adjusted *R*^2^	0.112	0.137	0.170	0.266	0.403
	△*R*^2^	0.116	0.026	0.173	0.093	0.137
Postgraduates	Gender	−4.521**	−4.368**	0.733	0.535	–0.698
	Exercise per week	2.300**	2.220**	−1.529**	−1.425**	–0.799
	Attitude to majors	9.430**	8.689**	−7.596**	−6.633**	−4.183**
	Problematic smartphone use		−0.263**		0.342**	0.268**
	Psychological capital					−0.282**
	*R*^2^	0.142	0.168	0.196	0.311	0.482
	*F*	38.608**	35.338**	56.922**	78.802**	130.036**
	Adjusted *R*^2^	0.138	0.163	0.193	0.307	0.479
	△*R*^2^	0.142	0.02	0.196	0.115	0.171

*p < 0.05, **p < 0.01.

**TABLE 4:** The effect of problematic smartphone use and psychological capital on three dimensions of learning burnout.

**Education background**	**Variables**	**Depression**			**Improper behavior**			**Low personal accomplishment**		
		**Block1 (β)**	**Block2 (β)**	**Block3 (β)**	**Block1 (β)**	**Block2 (β)**	**Block3 (β)**	**Block1 (β)**	**Block2 (β)**	**Block3 (β)**
Un	Gender	−1.050**	−0.989**	−0.970**	−1.310**	−1.276**	−1.257**			
	Residence							–0.426	–0.426	–0.365
	Exercise per week	−0.952**	−0.850**	−0.624**	−1.127**	−1.069**	−0.858**	–0.394	–0.362	–0.113
	Attitude to majors	−2.880**	−2.496**	−1.804*	−1.569**	−1.354**	−0.706**	−2.170**	−2.040**	−1.272**
	Problematic smartphone use		0.173**	0.151**		0.097**	0.076**		0.058**	0.034**
	Psychological capital			−0.095**			−0.089**			0.106**
	*R*^2^	0.135	0.247	0.309	0.108	0.167	0.256	0.138	0.164	0.320
	*F*	39.823**	62.890**	68.400**	31.022**	38.300**	52.710**	40.905**	37.531**	71.941**
	Adjusted *R*^2^	0.131	0.243	0.304	0.105	0.162	0.251	0.135	0.160	0.315
	△*R*^2^	0.135	0.112	0.062	0.108	0.058	0.090	0.138	0.026	0.156
Pos	Gender							–0.173	–0.236	−0.610*
	Exercise per week	−0.671**	−0.608*	–0.352	−0.626**	−0.588**	−0.378*	−0.638**	−0.605**	−0.415*
	Attitude to majors	−3.485**	−2.966**	−2.065**	−1.794**	−1.482**	−0.741**	−1.802**	−1.493**	−0.749**
	Problematic smartphone use		0.182**	0.155**		0.110**	0.087**		0.110**	0.087**
	Psychological capital			−0.101**			−0.083**			−0.086**
	*R*^2^	0.159	0.287	0.376	0.095	0.182	0.303	0.095	0.187	0.309
	*F*	66.046**	93.877**	105.091**	36.678**	53.277**	76.114**	24.540**	40.114**	62.342**
	Adjusted *R*^2^	0.156	0.284	0.372	0.092	0.182	0.299	0.091	0.182	0.304
	△*R*^2^	0.159	0.128	0.089	0.095	0.091	0.118	0.095	0.092	0.122

Un, undergraduates; Pos, postgraduates.*p < 0.05, **p < 0.01.

### Mediating Effect of Psychological Capital

As shown in Tables 3, 4, in undergraduates, the effect (β) of problematic smartphone use on learning burnout (depression, improper behavior, and low personal accomplishment) in step 3 (0.261, 0.151, 0.076, and 0.034) were smaller than that in step 2 (0.328, 0.173, 0.097, and 0.058); in postgraduates, the effect (β) of problematic smartphone use on learning burnout (depression, improper behavior, low personal accomplishment) in step 3 (0.268, 0.155, 0.087, and 0.087) were also smaller than that in step 2 (0.342, 0.182, 0.110, and 0.110); indicating psychological capital mediated this relationship in both undergraduates and postgraduates.

**FIGURE 2:**
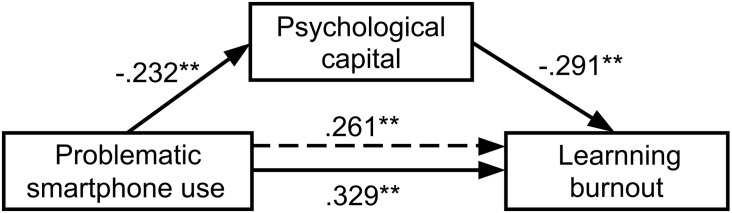
Models of the mediating effect of psychological capital between problematic smartphone use and learning burnout in medical undergraduates; ^∗^*p* < 0.05, ^∗∗^*p* < 0.01.

**FIGURE 3:**
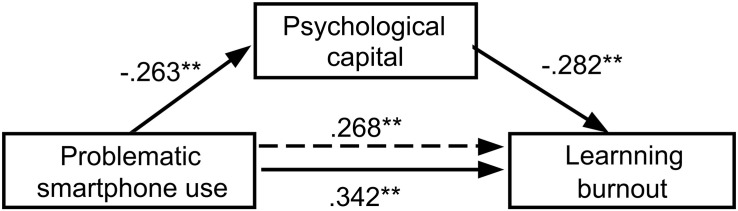
Models of the mediating effect of psychological capital between problematic smartphone use and learning burnout in medical postgraduates; ^∗^*p* < 0.05, ^∗∗^*p* < 0.01.

The bootstrap method was used to examine the significance of the mediating effect of psychological capital. As shown in Table 5, all the 95%bootCI did not contain 0. Therefore, the mediating effects of psychological capital between problematic smartphone use and learning burnout (depression, improper behavior, and low personal accomplishment) in both undergraduates and postgraduates were significant. The detailed models results are shown Table 5. We presented the main contents of Table 5 in the form of figure. As shown in Figures 2, 3; for medical undergraduates, the direct effect and total effect of problematic smartphone use on learning burnout was 0.261 and 0.329, respectively, psychological capital partially mediated this relationship, and this indirect effect was 0.068, constituting 20.67% of the total effect; for medical postgraduates, the direct effect and total effect of problematic smartphone use on learning burnout was 0.268 and 0.342, respectively, psychological capital partially mediated this relationship, and this indirect effect was 0.074, constituting 21.64% of the total effect.

**TABLE 5:** The significance of the mediating effect of psychological capital.

**Education Background**	**Path**	**a**	**b**	**c**	**c′**	a*b	**95%boot CI**
Undergraduates	PSU→PC→LB	−0.232**	−0.291**	0.329**	0.261**	0.068	0.036−−0.094
	PSU→PC→Depression	−0.232**	−0.095**	0.172**	0.151**	0.022	0.023−−0.068
	PSU→PC→IB	−0.232**	−0.089**	0.097**	0.076**	0.021	0.028−−0.078
	PSU→PC→LPA	−0.232**	−0.106**	0.058**	0.034**	0.025	0.038−−0.100
Postgraduates	PSU→PC→LB	−0.263**	−0.282**	0.342**	0.268**	0.074	0.039−−0.110
	PSU→PC→Depression	−0.268**	−0.101**	0.182**	0.155**	0.027	0.028−−0.082
	PSU→PC→IB	−0.268**	−0.083**	0.110**	0.097**	0.022	0.032−−0.095
	PSU→PC→LPA	−0.263**	−0.094**	0.050**	0.025**	0.025	0.041−−0.120

*PSU, problematic smartphone use; PC, psychological capital; LB, learning burnout; IB, improper behavior; LPA, low personal accomplishment; a, the effect of problematic smartphone use on psychological capital; b, the effect of psychological capital on learning burnout and it’s three dimensions; c, total effect; c′, direct effect; a^∗^b, indirect effect as well as the mediating effect of psychological capital in this relationship.*

## Discussion

Problematic smartphone use (Jing et al., 2016) and learning burnout (Liu et al., 2018) are pressing issues among Chinese medical students. In this study, 36.4% undergraduates and 27.6% undergraduates had a score higher than the mid-value (60), indicating that they had a high level of learning burnout. The objective of this study was to further investigate the relationship between problematic smartphone use and learning burnout, as well as the mediating effect of psychological capital in this relationship. Meanwhile, researches confirmed that psychological capital could be exploited like social resources (Luthans et al., 2006), and everyone has psychological resources at various levels, which can be the basis for intervention on dealing with learning burnout among Chinese medical undergraduates and postgraduates in the future.

This study found that learning burnout had significant differences between Chinese medical undergraduates and postgraduates, the level of undergraduates’ learning burnout was higher that of postgraduates, consisting with previous studies (Erdogan et al., 2012). A positive shift toward deep and strategic learning existed among postgraduates, which was not found among the undergraduates (Samarakoon et al., 2013). Moreover, the psychological and academic stress of undergraduates was significantly higher than that among graduates (Liang et al., 2009). Therefore, the undergraduates were more prone to learning burnout than the graduates. We analyzed undergraduate and postgraduate students separately because of their different psychosocial development stages.

Studies found that interest greatly influenced motivation and engagement (Renninger and Hidi, 2016); students who disliked their major would lose their motivation for study and would get burned out in the long run (Costa et al., 2012). Exercise was good for physical and mental health, which were more associated with the exhaustion component of burnout (Peterson et al., 2008) and also associated with a healthy mood and positive self-esteem (Barton et al., 2012), and these may help students reduce burnout (Wolf and Rosenstock, 2017). So we also controlled these factors in the analysis.

Then, this study confirmed the mediating effect of psychological capital between problematic smartphone use and learning burnout in both Chinese medical undergraduates and postgraduates. The results showed that problematic smartphone use of medical students was positively associated with their learning burnout (Bian et al., 2018); this result finds consonance in the literature. Problematic smartphone use indicates frequent usage and much time consumption (Liu et al., 2016), indicating that students spent less time in studying, thus negatively affecting academic performance gradually. Many studies have also confirmed the negative impact of problematic smartphone use on academic performance (Hawi and Samaha, 2016; Samaha and Hawi, 2016); academic performance is closely related to and can be considered an antecedent for learning burnout. Besides, studies have found that problematic smartphone use has a negative effect on people’s physical and mental health, such as headache, ear pain (Gao et al., 2017), sleep disorders (Liu et al., 2017), and suicidal ideation (Wang et al., 2014); these problems can consume so much energy of students that they can’t devote themselves to learning. Students who with problematic smartphone use are hard to disengage from smartphones quickly when stopping the use of it; thus, medical students cannot concentrate on their studies, resulting learning burnout. And the results of this study also showd psychological capital mediated this relationship; reasons might be found in the literature. The results are discussed as follows.

Psychological capital includes four dimensions: self-efficacy, hope, optimism, and resilience (Luthans et al., 2007a). Problematic smartphone use is identified as an impulse control disorder (Leung, 2008); it can make someone uncontrollably anxious and irritable because of the prolonged absence of the mobile phone (Chóliz, 2010), which could affect their conviction (or self-confidence) in their ability to mobilize themselves. As such, self-efficacy is destroyed (Luthans et al., 2008). Moreover, being addicted to mobile phones for prolonged periods can lead to mental health problems such as depression (Smetaniuk, 2014); this can have a negative impact on optimism and hope, which represent a “positive motivational state (Snyder et al., 1991).” Resilience represents relative protection for an individual against environmental stresses (Cadet, 2016). However, poor mental health such as depression and anxiety can be detrimental to resilience. Previous studies have shown that addiction behavior has an impact on resilience (Mak et al., 2018). Whereas resilience can protect one from Internet addiction (Robertson et al., 2018), self-efficacy may allow individuals to control themselves better (Luthans et al., 2008), and optimism and resilience are related to perseverance (Luthans et al., 2008). These positive psychological traits can lead to better self-control and prevent addiction behavior. In this study, problematic smartphone use of medical students had a negative impact on their psychological capital, but psychological capital worked as a protective factor against problematic smartphone use. Previous studies show that self-efficacy can compensate for increased learning burnout (Maricutoiu and Sulea, 2019). Students who had sufficient conviction and abilities to mobilize themselves would decrease improper behavior; resilience was associated with lower levels of learning burnout and emotional exhaustion (Rios-Risquez et al., 2016). Hope and self-efficacy may promote academic achievement (Feldman and Kubota, 2015), which is highly negatively correlated with learning burnout. Studies have also confirmed that psychological capital has a strong effect on burnout (Leon-Perez et al., 2016; Gong et al., 2019). This study demonstrated that psychological capital can mediate the relationship between problematic smartphone use and learning burnout, which is highly consistent with studies mentioned above. The results may have important implications in professional practice. Interventions for learning burnout can possibly conducted by exploiting the psychological capital of medical students in the future.

Nevertheless, the present study had some limitations. First, this was a cross-sectional study; any causal relationship based on the associations observed in our study should be inferred cautiously. Further research, such as cohort studies and intervention trials, is needed to increase its reliability. The researchers intend to elaborate further on the current findings in future research, for example, an intervention trial of learning burnout among medical students can be conducted by improving psychological capital. Second, the participants in this study were recruited from one city. This sample does not represent all medical students in China. However, Chongqing is known as a “miniature of China (National Bureau of Statistics of China, 2019),” and this is the only medical university in Chongqing. Future research is needed to show if these inferences can be applied to the medical students in other parts of China. Additionally, despite this study having a large sample covering almost all majors, the convenience cluster sampling method may have caused a selection bias and the external validation was not good enough. Third, the accuracy of self-reports is of concern and more measures must be taken to improve it in the future.

## Conclusion

Problematic smartphone use can directly predict learning burnout and their relationship was mediated by psychological capital in Chinese medical undergraduates and postgraduates. Therefore, Strategies to alleviating problematic smartphone use and enhance psychological capital in medical undergraduates and postgraduates may provide useful suggestions for future interventions on dealing with learning burnout in Chinese medical undergraduates and postgraduates.

## Data Availability Statement

The raw data supporting the conclusions of this article will be made available by the authors, without undue reservation.

## Ethics Statement

This study was approved by the Ethics Committee of Chongqing Medical University (2018015). Participants were fully informed regarding the survey prior to the participation. Completing and submitting the questionnaire was considered a proxy consent to participate. In this study, all participants were voluntary and the questionnaire was anonymous, we also used non-identifying codes, ensuring the confidentiality of the information.

## Author Contributions

CZ, XT, and FZ: conceptualization and methodology. CZ, GL, and ZF: data curation. CZ: formal analysis and writing – original draft. FZ: funding acquisition and writing – review and editing. CZ and ZF: investigation. CZ, GL, and FZ: project administration. GL, XT, and FZ: resources. GL: software. CZ and FZ: supervision. All authors contributed to the article and approved the submitted version.

## Conflict of Interest

The authors declare that the research was conducted in the absence of any commercial or financial relationships that could be construed as a potential conflict of interest.
